# Shaping Immune Responses in the Tumor Microenvironment of Ovarian Cancer

**DOI:** 10.3389/fimmu.2021.692360

**Published:** 2021-06-23

**Authors:** Xin Luo, Jing Xu, Jianhua Yu, Ping Yi

**Affiliations:** ^1^ Department of Obstetrics and Gynecology, The Third Affiliated Hospital of Chongqing Medical University, Chongqing, China; ^2^ Department of Hematology and Hematopoietic Cell Transplantation, City of Hope National Medical Center, Los Angeles, CA, United States; ^3^ Hematologic Malignancies Research Institute, City of Hope National Medical Center, Los Angeles, CA, United States

**Keywords:** ovarian cancer, tumor microenvironment, immune, genetic, epigenetic, cytokine

## Abstract

Reciprocal signaling between immune cells and ovarian cancer cells in the tumor microenvironment can alter immune responses and regulate disease progression. These signaling events are regulated by multiple factors, including genetic and epigenetic alterations in both the ovarian cancer cells and immune cells, as well as cytokine pathways. Multiple immune cell types are recruited to the ovarian cancer tumor microenvironment, and new insights about the complexity of their interactions have emerged in recent years. The growing understanding of immune cell function in the ovarian cancer tumor microenvironment has important implications for biomarker discovery and therapeutic development. This review aims to describe the factors that shape the phenotypes of immune cells in the tumor microenvironment of ovarian cancer and how these changes impact disease progression and therapy.

## Introduction

Ovarian cancer (OvCa) is the fifth most common cause of cancer death in women and has high mortality, with a 5-year overall survival rate of < 50% (1). Due to a lack of typical symptoms and effective early diagnostic measures, most patients are diagnosed at advanced stages (III and IV), when treatment options are limited (2, 3). Despite complete remission after debulking surgery combined with first-line chemotherapy, recurrence occurs in 70–80% of patients within 2–5 years, and chemotherapeutic resistance will eventually develop in all recurrent OvCa patients, leading to death (4, 5). The mechanism underlying recurrence and metastasis in OvCa is not clear, and may be related to changes in the immune system (6). The immune system consists of various cells and mediators, which protect against foreign pathogens and eliminate damaged cells to maintain tissue homeostasis (7). During tumor progression, immune cells often exhibit phenotypic and functional instability and transdifferentiate into different cell types or states, which can promote or inhibit tumor growth and metastasis (8, 9). Moreover, the infiltration of various immune cells into the tumor microenvironment (TME) is associated with clinical outcomes of OvCa (10). Therefore, understanding the cancer-associated changes in immune cells of the TME may clarify the mechanisms of OvCa pathogenesis and reveal novel biomarkers and therapeutic targets for OvCa (11).

The immune cell types in the OvCa TME and their functions have been extensively studied (12). However, the changes that occur in immune cells of the OvCa TME during cancer progression and how these insights might guide therapy are less clear. Here, we review how immune responses in the OvCa TME are shaped by the interactions between tumor cells and immune cells, which provides potential therapeutic targets and highlights the need for innovative therapeutic approaches.

## Infiltrating Immune Cells in the Ovarian Cancer Tumor Microenvironment

The tumor microenvironment (TME) refers to the niche, either primary or metastatic, where tumor cells interact with the host stroma including immune cells, endothelial cells, fibroblasts and metabolites. The important contribution of the TME to ovary cancer could manifest by the co-evolution of cancer and stromal cells which formed pre-metastatic niches and facilitated the peritoneal metastasis, such as neutrophil influxed into the omentum and extruded neutrophil extracellular traps (NETs), rendering the premetastatic omental niche conducive for implantation, was a prerequisite step for peritoneal metastasis in orthotopic ovarian cancer models (13); endothelial cells had activated Notch1 receptors (N1ICD) expression, facilitated peritoneal metastasis and associated with shorter survival in ovarian cancer-bearing mice, since sustained N1ICD activity induced EC senescence, expression of chemokines and the adhesion molecule VCAM1, promotes neutrophil recruitment and tumor intravasation (14).

The tumor immune microenvironment (TIME) is the immune contexture acting as a crucial orchestrator for cancer development, progression and metastasis, mainly composed with the infiltrated immune cells, their chemokines and cytokines (12). The relationship of TIME function and the clinical correlation were analyzed in ovarian carcinoma based on The Cancer Genome Atlas (TCGA) cohort, and four TIME molecular subtypes of the global immune-related genes were obtained, the high immune scoring subtype with the upregulated tumour-infiltrating immune cells had a high BRCA1 mutation, high expression of immune checkpoints, and optimal survival prognosis (15, 16). Cândido et al. evaluated the immune response patterns through analysis of type 1 (Th1), type 1(Th2), and type 17(Th17) cytokines in patients with epithelial ovarian cancer (EOC), and found higher levels of TNF-α/IL-4/IL-6/IL-10 in EOC patients compared to the control, IL-10 and TNF-α concentrations were higher in stage III/IV and associated with higher CA125, higher Th1 immune response was observed when the cytoreduction was considered optimal, while higher concentrations of Th2 cytokines were associated with unsatisfactory cytoreductive surgery and undifferentiated tumors (17).

**Figure 1:**
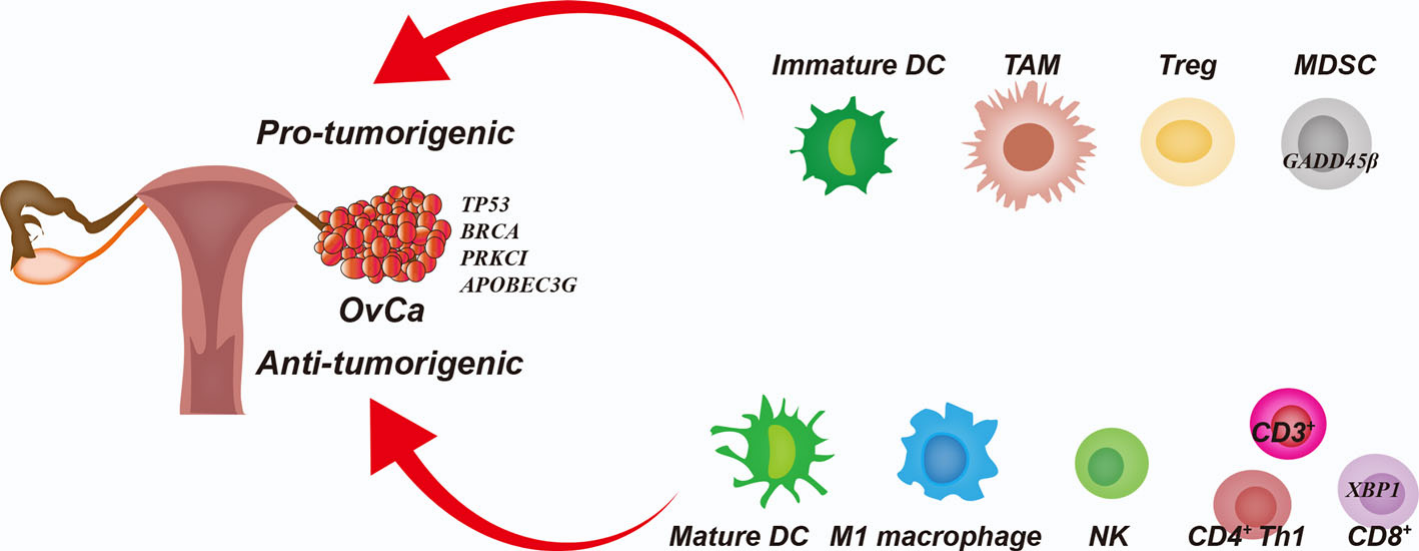
Interplay Among Cancer Cells and Immune Cells in the Ovarian Cancer Tumor Microenvironment. The immature dendritic cells (DCs), tumor-associated macrophages (TAMs), regulatory T cells (Tregs) and myeloid-derived suppressor cells (MDSCs) promote immunoresistance and therapeutic resistance in the ovarian cancer (OvCa) cells. Mature DCs, M1 macrophages, natural killer (NK) cells, and cytotoxic T lymphocytes (CTLs) inhibit tumor growth and increase the therapeutic susceptibility of OvCa cells.

The infiltrated immune cells can either limit or promote cancer development depending on the composition of immune cells and their phenotypic states. Notably, some infiltrated immune cells serve as tumor-associated immune cells, such as immature/tolerogenic dendritic cells (DCs), M2 macrophages, regulatory T (Treg) cells and, myeloid-derived suppressor cells (MDSCs). These cells maintain immune tolerance and suppress anti-tumor immunity, leading to OvCa therapeutic resistance (9). In contrast, mature DCs, M1 macrophages, natural killer (NK) cells, αβ T cells and γδ T cells can directly inhibit tumor growth or increase the susceptibility to checkpoint inhibitor therapies for OvCa (18, 19). Importantly, infiltration of CD4^+^ and CD8^+^ T cells into the tumor has been associated with improved overall and progression-free survival in OvCa patients (20). In **Figure 1**, we summarized the functions of immune cells in the OvCa TME.

The infiltrated immune cells functioned as a profound network regulating each other in the TIME. Several immunosuppressive cell types have been found migrating into OvCa tissues to promote immune escape by suppressing NK cells and cytotoxic T cells (21). For instance, M2 macrophages act as tumor-associated macrophages (TAMs) to subvert adaptive immunity and inflammatory circuits to promote tumor growth and progression (22). TAMs are the most abundant immune cells in advanced stage OvCa and foster tumor growth, invasion, angiogenesis, metastasis, and drug resistance (23). TAMs secrete IL10, IL6, TGF-β, CCL18, and CCL22, which attract regulatory T cells and promote differentiation of T cells towards the Th2 phenotype. IL10 and TGF-β also inhibit the cytotoxic activity of NK cells and cytotoxic T lymphocytes. Furthermore, CCL18 promotes T-cell anergy and unresponsiveness (24). In addition, Th17 cells and Tregs, which are subsets of CD4^+^ cells, maintain immunological self-tolerance and dampen anti-tumor activity in the TME, which is pro-tumorigenic in OvCa (22). A higher prevalence of Treg cells has been detected in tumors and malignant ascites of OvCa patients. The Treg cells directly inhibit other subsets of T cells by secreting the inhibitory cytokines IL-35, IL-10, and TGF-β or through binding checkpoint inhibitor receptors such as programmed cell death protein 1 (PD-1, also called PDCD1), cytotoxic T lymphocyte antigen 4 (CTLA4), and lymphocyte activation gene 3 (LAG3) (5). MDSCs are myeloid cells that suppress T cell responses and include myeloid progenitors and immature myeloid cells (25). MDSCs have been shown to accumulate in the circulation of cancer patients, and MDSC numbers generally correlate with an inferior prognosis (26). Advanced OvCa is associated with a myeloid bias that increases the frequencies of circulating granulocyte-monocyte progenitors (27). Tumor-derived factors, such as G-CSF (also known as CSF3), GM-CSF (also known as CSF2), and IL-6 drive this myeloid bias and increase the circulating and tumor-infiltrating MDSC population, which accelerates tumor progression by suppressing T cell responses and releasing metabolic factors (28). Furthermore, DCs are crucial for promoting and maintaining the anti-tumor immune response, which can coordinate the adaptive and acquired immune response to activate T cells (29).

## Genetic Alterations in OvCa Cells and Immune Cells in the TME

OvCa, especially high-grade serous OvCa (HGSOC), has been found to predominantly harbor mutations in *TP53*, loss of heterozygosity for *TP53*, mutations in *BRCA1/BRCA2*, loss of *PTEN*, and copy number abnormalities for other genes involved in homologous recombination (HR) DNA repair, resulting in high genomic instability (30, 31). OvCa cells with genomic instability also show has altered infiltration of immune cells in the TME (32). Non-homologous end-joining (NHEJ) occurs more frequently but may cause less severe mutations than HR and therefore is less studded in OvCa (30, 33).

*TP53* mutation is the most common event associated with poor clinical prognosis in HGSOC (34). The *TP53* status of the cancer cell has a profound impact on the immune response (35). TP53 controls the expression of multiple immunosuppression-associated proteins such as PD-L1 (also known as CD274), VISTA (also known as VSIR), NKG2D (also known as KLRK1), and FOXP3; loss or mutation of *TP53* in cancers changes cytokine secretion, resulted in reshaping the immune microenvironment to promote immune escape of cancer (36, 37). In OV-90 OvCa cell line, *TP53* loss promotes the recruitment of MDSCs and homing of the monocytes to the ascites through tumorigenic production of CCL2 (38). *TP53* deficiency in OvCa cells also increases the peripheral and intratumoral Treg populations, which are involved in suppressing effector T cells (39). Moreover, the interaction between TAMs and mutant *TP53* HGSOC promotes angiogenesis and epithelial-mesenchymal transition by increasing release of GATA3 exosome from TAMs, which is involved in the regulation of M2 macrophage polarization in the HGSOC TME (40). Taken together, these findings lead us to conclude that *TP53* mutation in OvCa cells acts as a critical player for the immunosuppressive effects of MDSCs, Tregs, and TAMs.

*BRCA1/BRCA2*-mutant tumors are often deficient in repairing double-stranded DNA breaks using HR, and these tumors exhibit increased therapeutic sensitivity to platinum-containing therapy and inhibitors of poly-(ADP-ribose)- polymerase (PARP) (41, 42). Somatic or germline *BRCA* mutations are present in approximately 25% of HGSOCs, which can give rise to a 10-fold increased risk of developing HGSOC (43). One study showed that HGSOCs with *BRCA1* disruption had more infiltration of CD8^+^ T cells in the TME than HR-proficient HGSOCs (44). This finding could be explained by the ability of BRCA1 to regulate cellular responses to inflammation, oxidative stress, and hypoxia, such as the direct role of BRCA1 in TNF-α and IL-1β signaling through NFκB, and interferon signaling through STAT1 (45). Moreover, survival analysis showed that *BRCA1/BRCA2*-mutant HGSOCs with high numbers of lymphocytes in the TME have a favorable prognosis (46). These findings document the relationship among *BRCA1/BRCA2*-mutation status, immunogenicity, and patient survival, suggesting that *BRCA1/BRCA2*-mutant HGSOCs may be more sensitive to immunotherapy than HR-proficient HGSOCs.

*PRKCI*, a gene encoding a serine-threonine kinase belonging to the atypical protein kinase C (aPKC) family, is located in the 3q26 locus, which is amplified in about 70% of HGSOC cases (44). Sharmistha et al. showed that *PRKCI* is amplified and overexpressed in OvCa and acts as an OvCa-specific oncogene. Furthermore, *PRKCI* overexpression in OvCa cells promoted nuclear localization of YAP1, leading to up-regulation of TNF expression, which then contributed to an immunosuppressive TME with an abundance of MDSCs and poor infiltration of cytotoxic T cells and NK cells (44). Thus, the PRKCI-YAP1 regulation of tumor immunity could provide an important window of diagnostic and therapeutic implications for OvCa (47).

In addition to somatic or germline mutations in OvCa cells, genomic amplifications are also found in the immune cells of the TME, which can regulate their phenotypes (48). APOBEC3G, one of the APOBEC family of antiviral DNA cytosine deaminases, is expressed broadly in human tissues (49). Leonard et al. showed that the expression levels of APOBEC3G are surprisingly high in cytotoxic (CD8A) and helper T (CD4^+^) lymphocytes in HGSOC and correlate positively with improved HGSOC patient outcomes (50). Engineering T cells with boosted APOBEC3G could be interesting to as a cellular immunotherapy against HGSOC. Unlike APOBEC3G, which confers immunosensitivity, elevated *GADD45B* expression confers poor clinical outcomes in most human cancers. *GADD45B* is an important myeloid-intrinsic factor for proinflammatory macrophage activation and the immunosuppressive activity of the TME, which restricts CD8^+^ T-cell trafficking into tumors (51). To explore the function of *GADD45B* in OvCa, Daniela et al. performed flow cytometry analysis of an OvCa allograft mouse model and found that conditional knockout of *GADD45B* in myeloid cells restores proinflammatory TAM activation and intratumoral CD8^+^ T-lymphocyte infiltration, resulting in reduced tumor growth (51). Moreover, a study revealed that upregulation of *XBP1* in CD4^+^ and CD8^+^ T cells isolated from OvCa specimens was associated with decreased infiltration of T cells into tumors and with reduced *IFNG* mRNA expression. *XBP1*-deficient T cells in the metastatic OvCa milieu exhibited global transcriptional reprogramming and improved effector capacity (52). Accordingly, mice that bear OvCa and lack *XBP1* selectively in T cells demonstrate superior anti-tumor immunity, delayed malignant progression, and increased overall survival; interestingly, the role of *XBP1* in NK cells may be opposite (53). Targeting *XBP1* may help to restore the metabolic fitness and anti-tumor capacity of T cells in cancer hosts (52). Therefore, all three genes as new candidate biomarkers for effective T-cell responses and provide potential enhancers of cellular immunotherapy for OvCa.

These data show that genetic alterations, which cause phenotypic changes both within the OvCa cells and in the immune cells of the TME, can impact immune cell infiltration and cancer prognosis. These genetic alterations are summarized in **Table 1**.

**Table 1:** Genes regulate immune system in OvCa.

Cell type	Gene alterations	Pathogenetic role	Ref
OvCa	TP53 deficiency	Increases MDSCs, Tregs and TAM populations	(39, 40, 54)
	BRCA mutation	Increases infiltration of CD4^+^ and CD8^+^ T cells	(45, 46)
	PRKCI amplification	Enhances MDSCs and reduces CD8^+^ T cells and NK cells infiltration	(44)
	APOBEC3G high level	Increases T cell infiltration	(50)
MDSCs	GADD45β deletion	Restores proinflammatory TAM activation and CD8^+^ T cells infiltration	(51)
T cells	XBP1 deficiency	Restores the metabolic fitness and anti­tumour capacity of T cells	(52)

OvCa, ovarian cancer; MDSCs, myeloid-derived suppressor cells; DCs, dendritic cells; TAM, tumor-associated macrophage; Treg, regulatory T; NK, natural killer.

## Epigenetic Effects of Noncoding RNAs in the OvCa TME

There is increasing evidence that epigenetic regulation by noncoding RNAs (ncRNAs) plays an important role in OvCa by reprogramming the phenotypes of immune cells in the TME (55). ncRNAs have especially been linked to immunosuppressive activities such as TAM polarization, MDSC recruitment, Treg development, and functional defects in NK cells and cytotoxic T cells in the OvCa TME (24).

The term ncRNAs includes a range of epigenetic regulatory RNAs, such as microRNAs (miRNAs) and long non-coding RNAs (lncRNAs) (56). ncRNAs mediate many fundamental cellular processes, such as development, differentiation, proliferation, transcription, post-transcriptional modifications, apoptosis, and cell metabolism (57). Recently, it was discovered that the expression of most ncRNAs is perturbed in cancer, and these up- or down-regulated ncRNAs are significantly correlated with numbers and types of immune cell infiltration in TME (58). Xu and colleagues identified miR-424(322) as a negative regulator of several mRNAs encoding immune regulatory proteins, including the T cell inhibitory ligands PD-L1and CD80, in chemoresistant OvCa cells (59). High levels of miR-424(322) in tumors are correlated with improved progression-free survival and, in a syngeneic OvCa mouse model, overexpression of miR-424(322) in the OvCa cells increased the number of cytotoxic CD8^+^ T cells and decreased the number of MDSCs and Tregs in the TME, reduced tumor growth, and enhanced the efficacy of chemotherapy (59). Moreover, Xie et al. found that miR-20a is overexpressed in human OvCa tissues and enhances long-term cellular proliferation and invasion capabilities by suppressing NK cell cytotoxicity through directly binding 3’-untranslated region (3’UTR) of MICA/B mRNA and downregulating its expression on the membrane of OvCa cells. MICA/B are ligands of the natural killer group 2 member D (NKG2D) receptor found on NK cells, γδ^+^ T cells and CD8^+^ T cells (60). The reduction of membrane-bound MICA/B proteins allows OvCa cells to evade immune-mediated killing (60). Furthermore, a study by An and Yang investigated the role of miRNAs in immune cells and indicated that miR-21 in macrophages could modulate M0 polarization into M2 by increasing the expression of M2 macrophage markers CD206 and IL-10, and decreasing the expression of M1 macrophage markers iNOS and TNF-α. Then, co-cultured M2 macrophages with miR-21 overexpression and OvCa cells found that M2 macrophages promote the chemoresistance of OvCa by activating PI3K/AKT signaling of tumor cells (61). Another miRNA with an inhibitory effect on polarization of M2 macrophages is miR-217. Transfection of OvCa cells with miR-217 suppresses expression of the secreted factor IL6, which attenuates M2 macrophage polarization through JAK/STAT3 signaling (62). In addition, it has been reported that lncRNAs are correlated with reprogramming of immune cells in OvCa. In a study by Shang et al., the authors found that the lncRNA HOTTIP was highly expressed in OvCa tissues, and overexpressing HOTTIP in OvCa cells promoted the expression of IL6 by binding to JUN. IL6 secretion then conferred PD-L1 expression on neutrophils, reduced CD3^+^ T cell proliferation, and reduced response to tumor immunotherapy (63). In another study, Colvin et al. revealed that high MIR155HG expression in cancer-associated fibroblasts (CAFs) in OvCa patients was associated with higher infiltrates of immune cell subsets, including CD8^+^ T cells, CD4^+^ memory activated T cells, follicular helper T cells, γδ^+^ T cells, M1 macrophages, and eosinophils, and with longer survival (64). A functional RNA co-expression enrichment analysis revealed that the Gene Ontology terms for RNAs co-expressed with MIR155HG could be grouped into categories associated with T cell activation, antigen processing and presentation, leukocyte migration, and activation of an immune response. A similar analysis revealed that the RNAs co-expressed with MIR155HG included Kyoto encyclopedia of genes and genomes (KEGG) pathways related to immune diseases and the immune system, suggesting a role for MIR155HG in regulating the immune microenvironment (64). However, the specific mechanisms and cells involved remain unknown.

One important aspect to consider in the regulatory role of miRNAs in the TME is that miRNAs can be transported beyond their cells of origin. Indeed, miRNAs can be transported inside extracellular vesicles (EVs) and delivered to recipient cells, regulating their biological functions (65). This miRNA-mediated cell-to-cell communication represents active crosstalk involving multiple cellular components of the TME, which include cancer cells, mesenchymal stromal cells, CAFs, endothelial cells, and immune cells. Interactions between OvCa cells and TAMs in promoting cancer progression have been reported to be mediated by miRNAs packaged in exosomes (66). One study reported that the exosomal miR-1246 derived from OvCa cells is abundantly expressed in OvCa exosomes and is taken up by M2 macrophages, which confers chemoresistance in OvCa cells through targeting Cav-1 mRNA of M2 macrophages and regulating p-gp interaction (67). Moreover, epithelial ovarian cancers (EOC) released exosomal miR-222-3p downregulates SOCS3 expression and activates STAT3 signaling pathways in macrophages, which induces polarization of the M2 phenotype and enhances the growth and metastasis of EOC cells (68). Similarly, the high expression of miR-940 in exosomes derived from EOC stimulated M2 phenotype polarization and promoted EOC proliferation and migration at the hypoxia environment (69). In addition, under the hypoxic condition, EOC cell-derived exosomes deliver miR-21-3p, miR-125b-5p and miR-181d-5p to macrophages and induce the polarization of M2 macrophages by regulating the SOCS4/5/STAT3 pathway at M0 macrophages, which promoted EOC cell proliferation and migration (70). Zhou et al. identified miR-29a-3p and miR-21-5p enriched in the exosomes derived from TAMs and led to imbalance of Treg/Th17 ratio to facilitate EOC progression and metastasis. Meanwhile, co-culture experiments involving TAMs and T cells or over-expressed the miR-29a-3p and miR-21-5p in CD4^+^ T cells also significantly increased the Treg/Th17 ratio in EOC. The mechanism suggests the supernatant release of two miRNA exosomes from TAMs in OvCa could target STAT3 of CD4^+^ T cells (22). Also, Czystowska et al. reported that small exosomes found in the ascites and plasma of OvCa patients contains ARG1 (arginase-1). ARG1-containing exosomes suppress proliferation of CD4^+^ and CD8^+^ T-cells *in vitro* and *in vivo* in OvCa mouse models by distributing ARG1 from tumor cells to antigen-presenting cells in secondary lymphoid organs. High expression of ARG1-containing exosomes contributes to tumor growth and tumor escape from the host immune system, and increased ARG1 activity in plasma is associated with worse prognosis in OvCa patients (71). Tumor-derived exosomes have also been reported to enhance immune suppression by promoting the differentiation of inhibitory immune cells, including TAMs and Treg cells.

**Figure 2:**
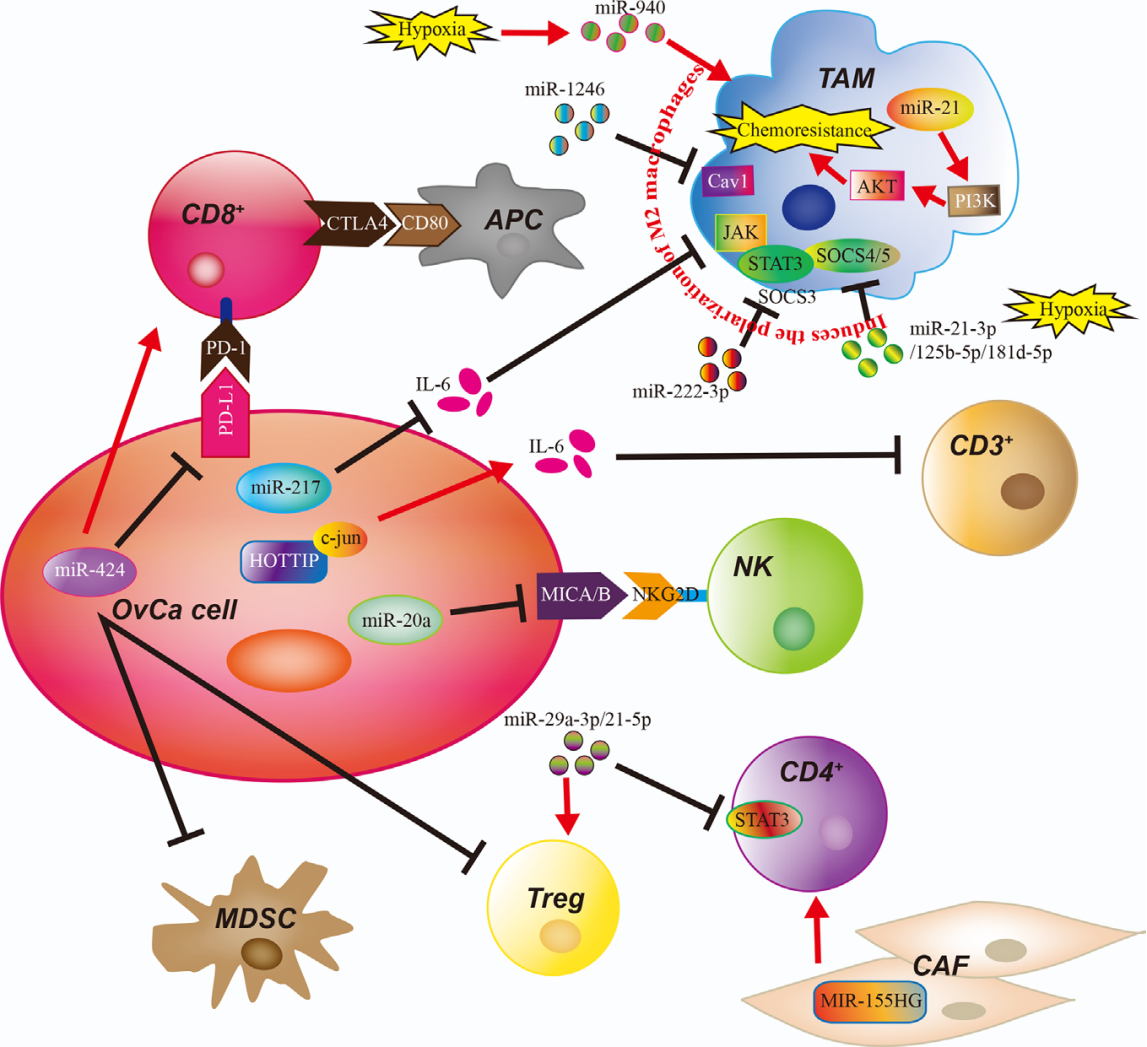
The Role of Noncoding RNAs in the Ovarian Cancer Tumor Microenvironment. The immune cells of the ovarian cancer (OvCa) tumor microenvironment are regulated by noncoding RNAs and exosomes containing micro-RNAs (miRs). APC, antigen-presenting cell; CAF, cancer-associated fibroblast; CD3^+^, CD3-expressing T cell, CD4^+^, CD4-expressing T cell; CD8^+^, CD8-expressing T cell; MDSC, myeloid-derived suppressor cell; NK, natural killer cell; TAM, tumor-associated macrophage.

The regulatory mechanisms linking OvCa and immune cell function *via* ncRNAs are detailed in **Figure 2** and **Table 2**. These findings underline the importance of continued research to identify ncRNA-modulated immune changes in the OvCa TME, as they may reveal novel insights, diagnostic strategies, and potential therapeutic targets for OvCa.

**Table 2:** miRNAs and lncRNAs regulate immune cells in OvCa TME.

Names	Function	Mechanism	Ref
miR-424(322)	Promotes proliferation CD8^+^ T cells and inhibition of MDSC and Treg cells	Regulates PD-L1/PD-1 and CD80/CTLA-4	(59)
miR-20a	Suppresses NK cell cytotoxicity	Binds MICA/B 3’-UTR	(60)
miR-21	Repolarizes M2 macrophages into M1	Activates PI3K/AKT signaling	(61)
miR-217	Suppresses M2 macrophage polarization	Inhibits IL-6/IL-6R/JAK2/STAT3 signaling	(62)
HOTTIP	Inhibits CD3^+^ T cell proliferation	Binds c-jun to promote the expression of IL-6	(63)
CAFs MIR155HG	Promotes higher infiltrates of immune cell subsets	No mention	(64)
miR-1246 exosome	Induces the polarization of M2 macrophages	Inhibits expression of Cav1	(67)
miR-222-3p exosome	Induces the polarization of M2 macrophages	Regulates SOCS3/STAT3 pathway	(68)
miR-940 exosome	Induces the polarization of M2 macrophages	Hypoxia induces the high expression of miR−940	(69)
miR-21-3p/125b-5p/-181d-5p exosome	Induces the polarization of M2 macrophages	Regulate SOCS4/5/STAT3 pathway	(70)
miR-29a-3p/21-5p exosome	Unbalance of Treg/Th17 cells	Suppresses expression of STAT3	(22)

OvCa, ovarian cancer; TME, tumor microenvironment; CAFs, cancer-associated fibroblasts; MDSCs, myeloid-derived suppressor cells; Treg, regulatory T; NK, natural killer.

## Regulation of Immune Cells in the OvCa TME *via* Cytokines

Although genetic and epigenetic factors regulate the immune cell phenotypes in the OvCa TME, the final effect on cell function depends on the expression of secreted factors (72). OvCa cells continuously secrete cytokines that regulate tumorigenicity in both autocrine and paracrine fashions. Cytokines mediate cell-to-cell interactions and regulate cell growth, differentiation, maturation, and immune response, participating in inflammatory reactions, wound healing, and tumor progression (73). Increasing evidence shows that immune cells reprogram their environments by interacting with cytokines, such as interleukins, chemokines, and growth factors (74).

Chronic inflammation is implicated in tumorigenesis and tumor progression. Cytokines mediate chronic inflammation and are involved in cancer progression by regulating the immune system (75). Increased levels of IL6 have been observed in many cancers, especially OvCa. In the OvCa TME, cancer cells secrete IL6, which inhibits the maturation of DCs and induces immunosuppressive alternatively activated TAMs, which compromise the activation of tumor-infiltrating T cells (76). On the other hand, IL6-producing MDSCs suppresses Th1 differentiation of CD4^+^ T cells, which decreases their ability to help CD8^+^ T cells and DCs, resulting in impaired adaptive immune responses against the development of OvCa (77). Moreover, a study by Isobe et al. found that M2-polarized TAMs were the primary IL6-secreting cells in peritoneal fluid from metastatic OvCa (77). IL6 induces JAK/STAT3 signaling by binding to the IL6 receptor (IL6R) to enhance OvCa cell growth and chemotherapy resistance (77). Also, multiple interleukins, including IL4, IL6, IL10, and IL13, are released from OvCa cells and other cells of the TME and strongly polarize TAMs into M2-like phenotypes in OvCa (24). In contrast, studies have found that NK cells preactivated briefly with IL2, IL15, and IL18 induce proliferation of NK cells to enhance IFNG production and NK-cell-mediated killing of OvCa *in vitro and in vivo* (78, 79). Significantly, IL12 secreted by genetically modified chimeric antigen receptor (CAR) T cells have also been shown to modulate the OvCa TME through multiple mechanisms, including reactivation of anergic tumor-infiltrating lymphocytes, inhibition of Treg-mediated suppression of effector T cells, and induction of Th1 CD4^+^ T cells to the tumor site (80). Furthermore, Ullah et al. demonstrated that IL1B-producing tumor cells mediate immune suppressive effects such as increased Tregs and diminution of NK and memory T cells by upregulating HLA-G expression through the NFKB pathway in OvCa (81). Overall, interleukins are responsible for the dysfunction of innate and adaptive immunity against OvCa, and an interleukin-targeting approach has achieved good results in animal experiments, indicating that interleukins might be therapeutically effective when combined with current immunotherapies (82).

Chemokines are the largest subfamily of cytokines and can be divided into CC chemokines, CXC chemokines, C chemokines, and CX3C chemokines, based on the location of the first two cysteine (C) residues. They play a critical role in tumor growth and metastasis as key mediators of the inflammatory response (83). A complex chemokine-signaling network has been proposed to influence the development and progression of OvCa by regulating the trafficking of infiltrating immune cells (83). Macrophage-derived chemokine CCL22 in the TME and malignant ascites facilitate Treg infiltration to the OvCa, which inhibits anti-tumor immunity (48). Katrina et al. showed that high expression of STAT1 and STAT1 target genes (CXCL9, CXCL10, and CXCL11) are strongly correlated with improved chemotherapy response in OvCa (84). The Th1 immune response recruiting NK cells and effector CD8^+^ T cells was enhanced by CXCL9, CXCL10, and CXCL11 derived from tumor cells, which can limit the diffusion and migration of OvCa cells (84). The chemokine landscape of OvCa is heterogeneous with high expression of lymphocyte recruiting chemokines (CCL2, CCL4, and CCL5) in tumors with intraepithelial T cells, whereas CXCL10, CXCL12, and CXCL16 are expressed quasi-universally, including tumors lacking intraepithelia T cells (85). Zsiros et al. found that dendritic cell (DC)-vaccine primed T cells expressed the cognate receptors for the above chemokines that were strongly correlation with the presence of tumor-infiltrating CD8^+^ T cells in OvCa. Importantly, *Ex vivo* CD3/CD28 costimulation and expansion of vaccine-primed T cells upregulated CXCR3 and CXCR4, and enhanced their migration toward universally expressed chemokines in OvCa (85). Thus, vaccine primed and CD3/CD28 costimulated T cells can prepare for adoptive therapy to expand the available pool of tumor-reactive T cells in OvCa TME. Moreover, the intraepithelial tumor-infiltrating lymphocytes recruited by tumor chemokine CCL5 release IFN-γ to activate TAMs and DCs to secrete CXCL9, which in turn establishes a positive loop effectively amplifying T cell recruitment in EOC. CCL5 and CXCL9 co-expression reveals immunoreactive tumors with longer survival and response to checkpoint blockade, including OvCa (86). However, another study found that CCL5 expression in OvCa cancer stem cells recruited Tregs to promote immunoresistance and tumor metastasis *via* intercellular CCL5-CCR5 interactions, and co-culture with ovarian cancer cell lines induced the expression of MMP9 in Tregs, which promoted the invasion and metastasis of OvCa cells (87). Moreover, Taki et al. found that SNAIL (also known as SNAI1) expression in OvCa cells induces OvCa progression *via* upregulation of CXCR2 ligands (CXCL1 and CXCL2) and recruitment of MDSCs. *Snail* knockdown in mouse OvCa cells reduces the expression of the CXCL1/CXCL2 chemokines, which attract MDSCs to the tumor *via* CXCR2. Blocking CXCR2 inhibits MDSC infiltration and delays progression of Snail-high mouse tumors (88). Interestingly, Idorn et al. found that lentiviral transduction of tumor ascites lymphocytes (TALs) with chemokine receptor CXCR2 significantly increased migration of TALs towards rhIL8 and autologous ascites, which provides the proof of concept that engineering TALs with a chemokine receptor is feasible and can improve homing of transduced TALs towards the OvCa microenvironment (89). In brief, many chemokines are associated with OvCa by mediating immune responses that may favor or inhibit tumor progression.

STATs belong to a family of cytoplasmic transcription factors that communicate signals from the cell membrane to the nucleus (90). Upon the binding of cytokines or growth factors to cognate receptors on the cell surface, STATs are tyrosine phosphorylated, particularly by the JAK, ABL or SRC kinase families (91). The STAT family includes seven structurally and functionally related proteins: STAT1, STAT2, STAT3, STAT4, STAT5A, STAT5B, and STAT6. They have essential roles in fundamental processes, including sustaining proliferation, evading apoptosis, inducing angiogenesis, promoting invasion, and suppressing antitumor immunity (92). Each STAT protein appears to have distinct physiologic functions in the immune response of OvCa. STAT3 and STAT5 are known to bind to the promoter and increase the transcription of FOXP3 in CD4^+^ T cells; this expression is essential for the conversion of naive CD4^+^ T cells into Tregs in the OvCa TME (93). Thus, activation of STAT3 in CD4^+^ T cells generates an inflammatory environment around the OvCa, which promotes tumor growth by stimulating angiogenesis and suppressing anti-tumor response (90). In addition, ascites from OvCa patients polarized macrophages toward the M2 phenotype through STAT3 activation in OvCa cells (90). A study reported that when tumor supernatants from the epithelial OvCa cell lines OVCAR3, CAOV3, and SKOV3 were co-cultured with CD8^+^ T cells, STAT5 phosphorylation was reduced, which diminished CD8^+^ T cell proliferation (94). Moreover, STAT1 activation recruit CD8^+^ T cells at the site of induction by inducing the production of the chemokines CXCL9, CXCL10 and CXCL11 that bind to the common chemokine receptor CXCR3 in OvCa. High level of STAT1 in OvCa cells was significantly correlated with levels of CD8A transcripts from intratumoral CD8^+^ T cells and increased prognostic in patients with HGSOC (84). However, recent research found that OvCa patients with high intratumoral STAT1 activation exhibited poor prognosis compared with patients with low STAT1 activation *via* immunohistochemical analysis, indicating STAT1 may have a dual role in tumor development (95). Cytokines can transmit signals to STATs, and STATs can regulate the expression of cytokines by binding promoters, thus forming a circular pathway to promote OvCa immunosuppression and metastasis.

**Figure 3:**
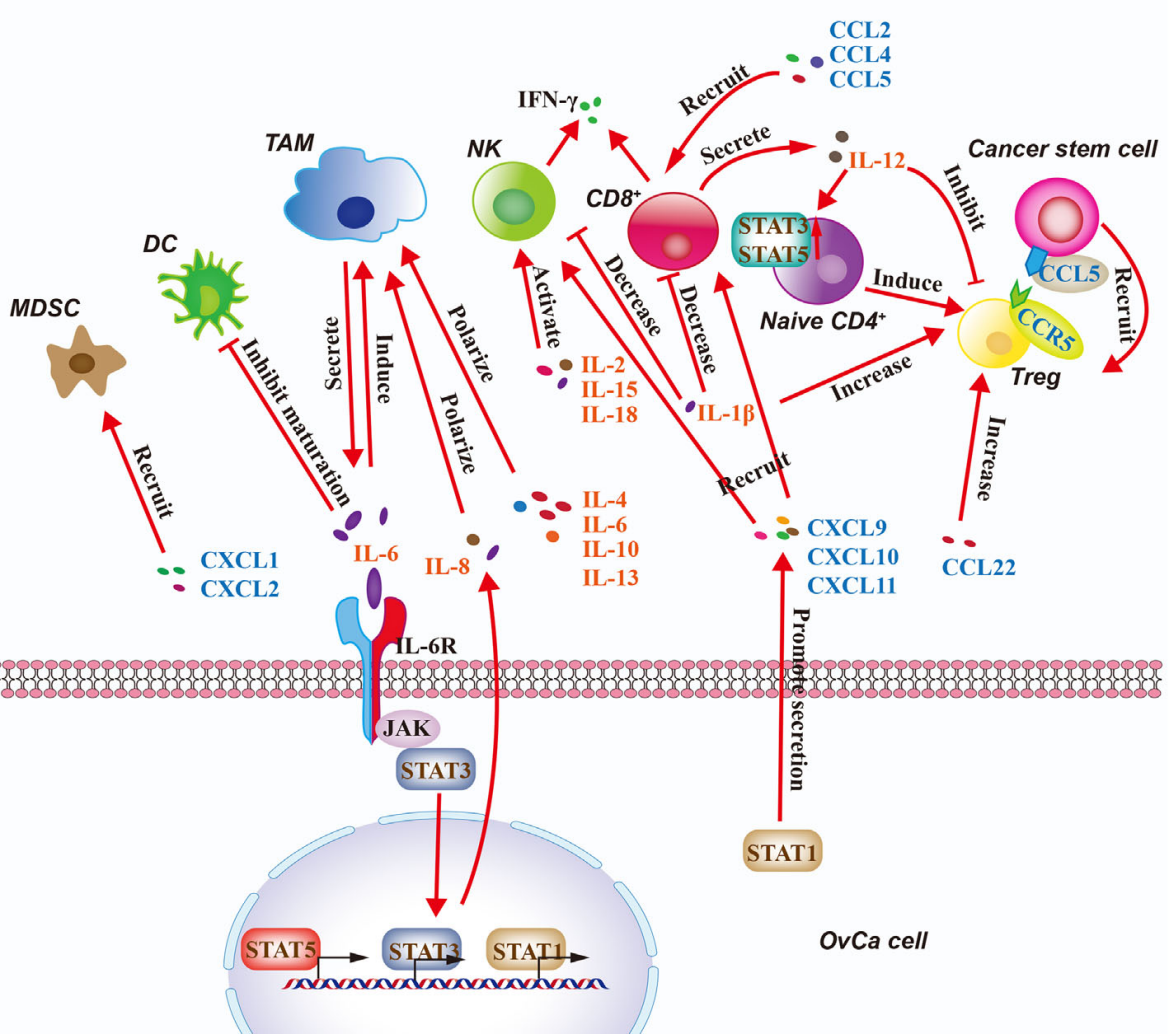
Regulation of the Immune Microenvironment in OvCa by Cytokine Signaling. Cytokine signaling pathways in the ovarian cancer (OvCa) tumor microenvironment include interleukins, chemokines, with intracellular regulation *via* STAT1/3/5. Immune cells and OvCa cells interact through cytokines and STATs to generate a comprehensive network at the tumor site. CSC, cancer stem cell; DC, dendritic cell; CD4^+^, CD4-expressing T cell; CD8^+^, CD8-expressing T cell; MDSC, myeloid-derived suppressor cell; NK, natural killer cell; TAM, tumor-associated macrophage; Treg, regulatory T cell.

Therefore, cytokine signaling components in the OvCa TME include interleukins, chemokines, and STATs. They play crucial roles in immune cell recruitment in the TME to influence OvCa clinical outcomes (96). Immune cells and OvCa cells interact through cytokines to generate a comprehensive network at the tumor site, which is responsible for the overall progression of the tumor (**Figure 3**). The roles of cytokines in OvCa are summarized in **Table 3**.

**Table 3:** Cytokines and STATs involved in regulating immune cells of OvCa.

Immune cells	Interleukin	Chemokine	STATs
DCs	IL-6	NA	STAT3
Macrophages	IL-4/-6/-10/-13	CCL5	STAT3
MDSCs	NA	CXCL1/2	NA
NK cells	IL-2/-15/-18 and IL-1β	CXCL9/10/11 and CCL2/4/5	NA
CD4^+ Foxp3-^ T cells	IL-6/-12	CXCL9/10/11	STAT3
CD8^+^ T cells	IL-6	CXCL9/10/11	STAT1/3/5
Tregs	IL-12	CCL5/22, CXCL2	STAT3/5
Ref	(77–82)	(85–90)	(81, 91, 94–96)

DC, dendritic cell; OvCa, ovarian cancer; MDSC, myeloid-derived suppressor cell; Treg, regulatory T cell; NK, natural killer; STAT, signal transducer and activator of transcription.

## Preclinical and Clinical Application: Targeting Immune Responses for the Treatment of OvCa

Due to nearly 75% of OvCa patients are diagnosed at a late stage with widespread intra-abdominal metastasis, cytoreductive surgery and primary chemotherapy with platinum agent and taxane have not been very effective (97). The majority (over 70%) of patients will relapse, with 5-year survival rates of approximately 30% and the proportion of patients who remain cancer-free at 10 years is less than 15% (98). Based on the detailed evidence with existing studies, certain disease mechanisms can be chosen as treatment targets. Currently, several targeted drugs have been approved by the Food and Drug Administration (FDA) and some of them are being tested in randomized controlled trials including mutant gene repairers, immune checkpoint inhibitors, Poly (ADP-ribose) polymerase (PARP) inhibitors and angiogenesis inhibitors (99). Despite these effects were promising, these targeted drugs were difficultly adopted as first-line therapy, because that remains poor response and increased risk of drug toxicity and death (100). For example, the response rate of anti-PD1 and anti-CTLA-4 treatments in OvCa clinical trial is 10-20%, because the majority of patients have high PD-L1 expression or lack T cells with appropriate anti-tumor reactivity (101). PARP inhibitors is only limited to populations with *BRCA* mutation associated OvCa with the FDA approval and the efficacy is somewhat limited (102). Therefore, novel clinical biomarkers and new therapeutic strategies should be developed.

In OvCa, the tumor mutational burden (TMB) is a positive relationship with the presence of neoantigens on cancer (103). Vaccine-induced tumor-associated antigen-specific immune response that could eliminate OvCa at its earliest stages is an attractive notion. The vaccine group with notable amplified T cell response and prolonged survival compared to a mock vaccine, but the heterogeneous character of OvCa makes it difficult to select an appropriate antigen to candidate as vaccine (104). Moreover, epigenetic therapies for OvCa can reinvigorate the antitumor immunity in tumor cell lines and mouse models (105). In particular, DNMT and HDAC inhibitors can reverse immune evasion and sensitize to subsequent immune checkpoint blockade by inducing an interferon response *via* upregulation of surface tumor antigens and key immunomodulatory proteins (105). Stone et al. demonstrated that the activation of type I interferon signaling in response to DNMT inhibitor 5-azacytidine (AZA) was a key requirement for efficient stimulation of CD45^+^ immune cells, CD8^+^ cytotoxic T cells and NK cells, restriction of macrophages and MDSCs in the OvCa (106). In support, Sara et al. demonstrated the enhanced expression of cancer-testis antigens and class I major histocompatibility complex (MHC)-encoded molecules in OvCa cells that were treated with DNMT inhibitors and subsequently increased infiltration of CD8^+^ cytotoxic T cells, NK cells, and NKT cells and decreased infiltration of MDSCs and PD-1hi CD4 T cells in OvCa microenvironment (107). Additionally, reports have shown that HDAC inhibitors suberoylanilide hydroxamic acid (SAHA) can also inhibit OvCa growth and enhance the host immune response against cancer cells *via* the suppression of Tregs and FoxP3 expression, upregulation of NK cell-activating ligands, MHC molecules (class I and II), enhancement of NK cell and CD8^+^ T cell cytotoxicity and production of proinflammatory cytokines (108). However, clinical trials with single-agent epigenetic therapy demonstrated disappointing effects in OvCa and showed severe toxicity profile of these drugs including fatigue, vomiting, and neutropenia (105). In addition, cytokine therapy is easily translated with small molecule drugs that has advantages in clinical treatment (109). Indeed, pre-clinical trials revealed that anti-IL-6 monoclonal antibody exerted anti-tumor efficacy for OvCa patients (110). However, therapies targeting cytokines also show limitations in treating OvCa. In phase I/II trial, anti-cytokine drugs had not improved response and clinical benefits in advanced OvCa patients (111). These drug therapies are all clearly listed in **Table 4**, which also shows the importance of the targeted mechanism.

**Table 4:** Major selected drugs and therapy regimens in clinical studies for ovarian Cancer.

Therapeutic regimen	Drug name	Function	Clinical trial identifier	Ref
Targeted therapy	Avelumab	Blocks PD-L1	NCT01772004	(112)
	Nivolumab	Blocks PD-1	UMIN000005714	(113)
	Ipilimumab	Blocks CTLA-4	NCT01611558	(114)
	APR-246	Binds TP53 *via* cysteine 277	NCT03268382	(115)
	Olaparib	Prevents the cell from repairing single-stranded DNA breaks	NCT0247764	(116)
	Bevacizumab	Inhibits VEGF	NCT01305213	(117)
	Aflibercept	Inhibits VEGF and PlGF	NCT00327444	(118)
	Apatinib	Inhibits VEGFR2	NCT02867956	(119)
	catumaxomab	Inhibits the EpCAM	NCT00326885	(120)
Vaccine	MUC1-vaccine	Targets MUC1	NCT01068509	(10)
	NY-ESO-1 vaccine	Targets NY-ESO-1	NCT00616941	(121)
Epigenetic therapy	DNMTi (AZA)	Removes methylation from ERVs	NCT01897571	(122)
	HDACi (SAHA)	Upregulates the expression of ERVs	NCT02915523	(105)
Cytokine therapy	Siltuximab	Inhibits IL-6	NCT00841191	(111)
	Tocilizumab	Inhibits IL-6 receptor	NCT01637532	(123)
	Carlumab	Inhibits CCL2	NCT00992186	(124)

VEGF, vascular endothelial growth factor; PlGF, placental growth facto; EpCAM, epithelial cell adhesion molecule; DNMTi, DNA methyltransferase inhibitor; HDACi, histone deacetylase inhibitor; AZA, 5-azacytidine; SAHA, suberoylanilide hydroxamic acid; ERVs, endogenous retroviruses.

Mono-immunotherapy has not achieved satisfactory clinical results in the most HGSOC patients, but a positive effect has been observed after combined therapy (125). Recent studies have demonstrated that poly (ADP-ribose) polymerase inhibitors (PARPis) exhibit anti-tumor immunity that occurs in a stimulator of interferon genes (STING)-dependent manner and is augmented by immune checkpoint blockade (126). In OvCa, combined PARPi and anti-PD-1/PD-L1 therapy has yielded encouraging preliminary results in two early-phase clinical trials (127). Moreover, combining PD-1 blockade with a single dose of the cancer vaccines GVAX or FVAX resulted in enhanced clonal expansion of antigen-specific CD8^+^ T cells and tumor control in OvCa (8). Similarly, PD-1 blockade and IL-10 neutralization were inefficient as monotherapies, but the combination of these two led to improved survival and delayed tumor growth in OvCa. This survival benefit was accompanied by augmented anti-tumor T and B cell responses and decreased infiltration of immunosuppressive MDSCs (128). Furthermore, studies showed that using DNMT or HDAC inhibitors in combination with anti-PD-1 or anti-CTLA-4 therapy enhances the antitumor immune response, reduces tumor burden and improves treatment outcomes in OvCa mouse models compared to each drug alone (105). In addition, recent research found that the microelement manganese (Mn2^+^) promoted DC and macrophage maturation and tumor-specific antigen presentation, augmented CD8^+^ T cell and NK cell activation and increased the number of memory CD8^+^ T cells in a STING-dependent way. Patients with platinum and/or anti-PD-1 antibody-resistant metastatic OvCa achieved partial response following the administration of Mn2^+^ (129). The balance between immune-stimulating and immunosuppressive factors in the TME has revealed a complex regulatory mechanism in OvCa. Thus, it has been broadly considered that combination cancer immunotherapy vs. monotherapy is the future direction of OvCa treatment, such as PARPis combined with immunotherapy, angiogenesis inhibitors combined with PARPis or immunotherapy (129).

**Figure 4:**
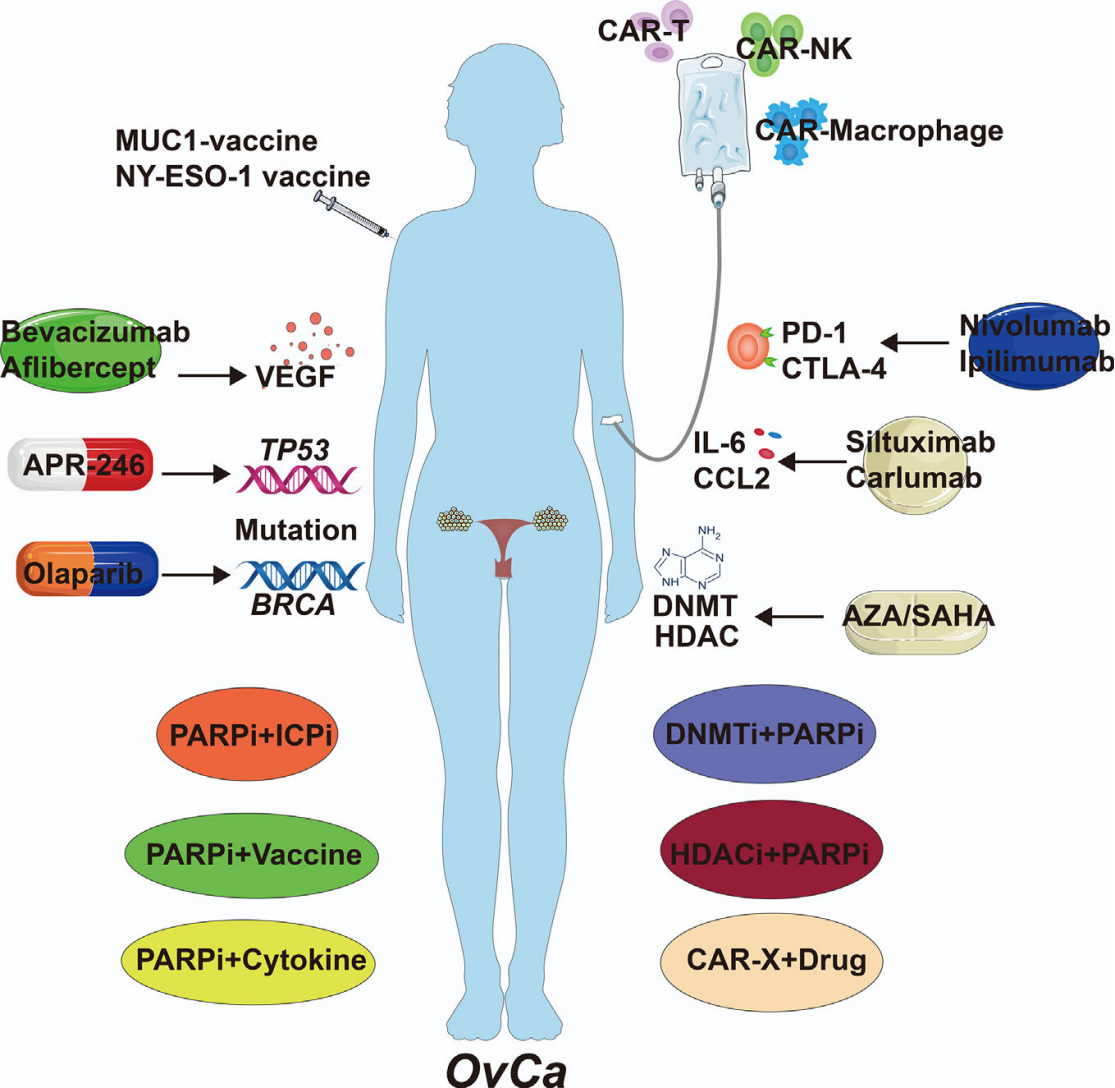
The Clinical Therapies of OvCa. Immunological therapies of OvCa include drug, cell and combination therapy. VEGF, vascular endothelial growth factor; DNMT, DNA methyltransferase; HDAC, histone deacetylase; AZA, 5-azacytidine; SAHA, suberoylanilide hydroxamic acid; CAR, chimeric antigen receptor.

The limitations of the drug therapies reviewed above in the treatment of OvCa prepare the groundwork for the use of novel immune cell therapies to treat this disease, either innate or adaptive immune cell therapies. Adoptive cellular therapy (ACT) that ex vivo–induced antigen-specific immune cells are infused back to patients to overcome immunosuppression (130). The chimeric antigen receptor T (CAR-T) cell therapy is a potential strategy in adoptive antitumor treatment (131). Four CAR-T cell therapies have been approved by the FDA for lymphoblastic leukemia, but neither approach applies to OvCa (132). Recently, FDA approves Abecma (idecabtagene vicleucel) as the first B-cell maturation antigen (BCMA)-CAR T cell immunotherapy for the treatment of relapsed or refractory multiple myeloma, which led to objective responses in 72% of heavily treated patients (133). For OvCa patients, CAR-T cells targeting the CA-125 tumor antigen are being developed and have shown promise against human xenograft models and plans to evaluate their safety in in-human phase I clinical trials have been reported (134, 135). Moreover, CAR-T cell therapy for OvCa with other common target antigens include mesothelin (MSLN), HER2 and FRα, which proliferate steadily *in vivo* and accumulate specifically in tumor tissues to enhance the antitumor effect (135). Fang et al. generated CAR-T cells with *piggyBac* (PB) transposon vector encoding scFV for MSLN and full-length antibody for PD-1 (αPD-1-mesoCAR-T cells) that been used in patients with refractory OvCa combined with an anti-angiogenic drug, apatinib. The patient achieved partial response with inhibition of liver metastatic nodules and survived for 17 months and had mild side effects with only grade 1 hypertension and fatigue (136). CAR-T cells offer the promise of prolonged remission after a single infusion, but challenges include the need to wait for the patient’s own cells to be engineered ex vivo, the risk of cytokine storms and graft-*versus*-host disease, and high production costs (137, 138). On the other hand, NK cells do not require human leukocyte antigen (HLA) matching to a specific patient, it is feasible and safe to transfer cells across allogeneic barriers (139). Thus, NK cell lines or ex vivo-expanded NK cells from third-party donors could be used as “off-the-shelf” cellular therapies, with the potential for lower costs and shorter wait times (140, 141). Recently, CAR-NK92 cells targeting CD24 were shown to kill CD24-expressing OvCa cell lines *in vitro* by producing high levels of IFN-γ (142). With more *in vivo* experiments and clinical studies ongoing, NK cell therapies may achieve revolutionary advances in the treatment of OvCa (143–147). However, the source of the NK cells, as well as the persistence, expansion, homing, and trafficking of the NK cells after being transferred into the patient, are great challenges (148). In addition, CAR-macrophage (CAR-M) has been demonstrated antigen-specific phagocytosis and pro-inflammatory M1 polarization in vitro, which was able to cross-present antigen and activate T cells (149). Interestingly, there are now many ongoing clinical trials evaluating the effects of combinatorial immune checkpoint blockade (targeting either PD1 or PDL1) with CD19-targeted CAR-T cells, the early results suggest that combinatorial treatment is safe and has a low toxicity profile and prolonging T cell function and limiting exhaustion (150). Innovative approaches to increase trafficking and limit suppression by anti-inflammatory cytokines and cells in the TME are also in development (151). Overexpression of IL-7 and CCL19 in CAR-T cells increased infiltration of pro-inflammatory dendritic cells and T cells into solid tumor tissues and enhances tumor regression in mouse models (151). In human OvCa cells, the HDAC inhibitor valproate (VPA) was reported to upregulate various NKG2DLs in human OvCa cells and enhance their susceptibility to CAR T cell-mediated attack (152). Adoptive transfer of NY-ESO-1–specific CD8^+^ TCR gene-engineered T cells, in combination with the demethylating agents decitabine and SGI-110, elicited synergistic inhibition of tumor growth, curing a fraction of OvCa mice (153). Thus, the combination of adoptive cell therapy and drug therapy has shown promising results as a novel treatment strategy for OvCa patients. A limitation of genetically reprogrammed immune cell therapeutics is the use of viral vectors that have expensive and long production times for clinical use (151). Researchers are developing a new non-viral method for delivering DNA sequences to primary immune cells and exploring the proper cocktail of cytokines for growth conditions of immune cells (151). Finally, we describe the mono-therapy and combination therapy in OvCa patient (**Figure 4**).

## Conclusions and Perspectives

The immune system plays an important role in the occurrence and development of OvCa, and immune dysregulation can lead to immune escape and resistance (154). Studies of immune cells in the OvCa TME have focused on T cells, DCs, MDSCs, macrophages, NK, and γδ T cells, as well as B cells (9). The B cells, mature DCs along with NK cells and T cells, are recognized as the main effector cells of immunity, which suppress tumor progression by secreting immunoglobulins or perforin/granzyme, thereby promoting immune response, and killing cancer cells directly. However, some immune cells play immunosuppressive roles in the OvCa TME, such as immature DCs, Tregs, MDSCs, and M2 macrophages, which serve as immunosuppressive factors to inhibit the cytotoxic functions of NK and CD4^+^/CD8^+^ T cells (155). In this review, we mainly describe various factors that affect the phenotype of immune cells in OvCa, including transcriptional and post-transcriptional factors, as well as cytokine signals. The main genes that affect the phenotype of immune cells are those that are frequently mutated or amplified in OvCa. In addition to mutations in the tumor cells, mutations also accumulate in the immune cells themselves, especially myeloid cells. Furthermore, ncRNAs, including miRNAs and lncRNAs, regulate the activity of immune cells in OvCa by binding target genes (156). Many recent studies have shown that OvCa cells and TAMs can release miRNA exosomes, thereby regulating immune cell phenotypes. Finally, cytokine signaling components, including interleukins, chemokines, and STATs, often mediate the interaction between immune cells and tumor cells in the OvCa TME to regulate immune system reorganization. The immune cells can be regulated by many factors in the development of OvCa, and elucidating how these factors shape immunity in the TME should provide insight to develop novel therapeutics to treat OvCa. Aimed at the genomic instability in HGSOC, therapeutic drugs have been developed by targeting mutation of *TP53* and *BRCA* (105). Then, in our review, we found that *PRKCI, APOBEC3G, GADD45B* and *XBP1* also could be potential target for OvCa therapy, and their remarkable regulation of immune *in vitro* or *in vivo* has been confirmed. Moreover, ncRNAs are important to carcinogenesis of OvCa and regulation of immune system, but the therapeutic strategies focused on ncRNA are few studies. The prognosis of HGSOC is generally poor and mono-therapy often exerts low response rates and serious side effects. To broaden the clinical benefit and safety and minimize the therapeutic costs, cellular engineering therapies with NK cells and combination of different immunotherapies and/or chemotherapies are considered to be the future direction of OvCa treatment. However, the present clinical benefit is only available for a fraction of OvCa patients.

Understanding the precise cellular and molecular mechanisms is a critical task to further improve the current immunotherapies or develop new therapeutic avenues. Recent applications of single-cell RNA sequencing (scRNA-seq) in the TME have provided important insights into the biology of tumor-infiltrating immune cells, including their heterogeneity, dynamics, and potential roles in both disease progression and response to immunotherapies (157). ScRNA-seq has been used in a variety of tumor research, including OvCa (1). However, most of the single-cell studies focused on OvCa cells and malignant ascites, and just one study revealed the tumor immune phenotypes of OvCa (158–160). It is believed that there will be single cell research on immune cells of ovarian cancer in the near future, which will further reveal the causes of phenotypic changes of immune cells, and provide novel gene targets to pursue as well as promising gene-based biomarkers to stratify patients for clinical actions.

## Author Contributions

All authors listed have made a substantial and direct contribution to the work, and approved it for publication.

## Conflict of Interest

The authors declare that the research was conducted in the absence of any commercial or financial relationships that could be construed as a potential conflict of interest.
